# The Puzzling Unidimensionality of DSM-5 Substance Use Disorder Diagnoses

**DOI:** 10.3389/fpsyt.2013.00153

**Published:** 2013-11-25

**Authors:** Robert J. MacCoun

**Affiliations:** ^1^Goldman School of Public Policy and Berkeley Law, University of California at Berkeley, Berkeley, CA, USA

**Keywords:** dependence, DSM, diagnosis, psychometrics, addiction

## Abstract

There is a perennial expert debate about the criteria to be included or excluded for the DSM diagnoses of substance use dependence. Yet analysts routinely report evidence for the unidimensionality of the resulting checklist. If in fact the checklist is unidimensional, the experts are wrong that the criteria are distinct, so either the experts are mistaken or the reported unidimensionality is spurious. I argue for the latter position, and suggest that the traditional reflexive measurement model is inappropriate for the DSM; a formative measurement model would be a more accurate characterization of the institutional process by which the checklist is created, and a network or causal model would be a more appropriate foundation for a scientifically grounded diagnostic system.

## Introduction

Since the first Diagnostic and Statistical Manual of the American Psychiatric Association (DSM) appeared in 1952, there have been six revisions – roughly one revision every decade [DSM-II in 1968, DSM-III in 1980, DSM-III-R in 1987, DSM-IV in 1994, DSM-IV-TR in 2000, and DSM-5 in 2013; see Ref. (1)]. These manuals provide an evolving checklist of possible indicators of drug abuse and/or drug dependence, some subset of which will trigger a distinct categorical diagnosis. Because these diagnoses are seen as consequential for clinicians, clients, treatment facilities, third-party payers, and for the development of addiction science, the years preceding each revision always see a lively and vigorous debate among experts about which indicators of substance abuse and dependence – e.g., withdrawal, tolerance, cravings, legal problems – do or don’t belong in the checklist.

To an outside observer, the process can appear chaotic and as political as it is scientific. But somehow, the resulting checklist seems to have a noteworthy psychometric property. Using popular psychometric methods, it has been argued that the DSM diagnostic criteria for substance dependence (DSM-IV) or a substance use disorder (DSM-5) form a *unidimensional* scale – implying that they are tapping a single, coherent latent construct, either “substance abuse” (for a given substance), “substance dependence,” or in the newest iteration, the combined construct “substance use disorder” [e.g., (2–6)].

But there is something odd about this. If indeed the DSM criteria form a unidimensional construct, then there should be little reason to spend years debating specific items to include in the construct. Under the measurement model that characterizes most psychometric analyses of DSM data, these indicators should be roughly interchangeable, in the same way that different items on an attitude scale, vocabulary test, or personality trait inventory tap different manifestations of the same underlying construct. And the corollary observation is that if the criteria that get debated – withdrawal, tolerance, craving, and the like – are indeed conceptually and empirically distinct (as I think they almost certainly are), then the evidence for the unidimensionality of the DSM criteria is perhaps puzzling or even troubling, rather than reassuring.

This essay does not contend the DSM diagnostic criteria are foolish or meaningless, or that adopting them was a serious mistake by some criterion of harm to patients. Rather, I argue that (a) there is confusion about the underlying structure of the DSM substance-related diagnostic criteria, and (b) greater clarity might promote the development of better science, better practice, and better inputs to management and policymaking. These are analytic issues that deserve attention in the coming decade, in anticipation of the eventual next iteration, DSM-6.

## DSM-IV and DSM-5 Substance-Use Criteria

The DSM-IV (7) distinguished “substance abuse” from “substance dependence,” using a checklist of seven criteria for the latter (see Table 1). The DSM-IV was described in terms of two dimensions (abuse and dependence), but my comments about it (and its research literature) refer to the claim that the second factor, dependence, was unidimensional. The DSM-5 (1, 8) collapses the distinction between abuse and dependence, and calls for a “substance use disorder” diagnosis, triggered by any two or more of 11 criteria, with six or more indicating a severe case. This decision was apparently based in part on evidence that the abuse items and the dependence items formed a single dimension [e.g., (6)].

**Table 1:** **DSM-IV “substance dependence” and DSM-5 “substance use disorder” diagnostic criteria**.

Criterion	DSM-IV substance dependence	DSM-5 substance use disorder
Tolerance	✓	✓
Withdrawal	✓	✓
Taken more/longer than intended	✓	✓
Desire/unsuccessful efforts to quit use	✓	✓
Great deal of time taken by activities involved in use	✓	✓
Use despite knowledge of problems associated with use	✓	✓
Important activities given up because of use	✓	✓
Recurrent use resulting in a failure to fulfill important role obligations		✓
Recurrent use resulting in physically hazardous behavior (e.g., driving)		✓
Continued use despite recurrent social problems associated with use		✓
Craving for the substance		✓

## Alternative Psychometric Models for Latent Constructs

What would it mean for a list of such criteria to constitute a unidimensional latent construct? There are several alternative psychometric measurement models that can operationalize a latent construct. They quite literally imply different metaphysical assumptions – ontologically, what construct exists, and epistemologically, how to do we identify it? – but also different mathematical definitions. The discussion that follows gets slightly technical, and requires a few simple equations, but to keep things simple I assume there is only one latent construct (e.g., “dependence”) and that the terms in the model have unit weights (i.e., *w_i_* = 1 so that *w_i_X*_i_ = *X_i_*).

### Reflective models

**Figure 1:**
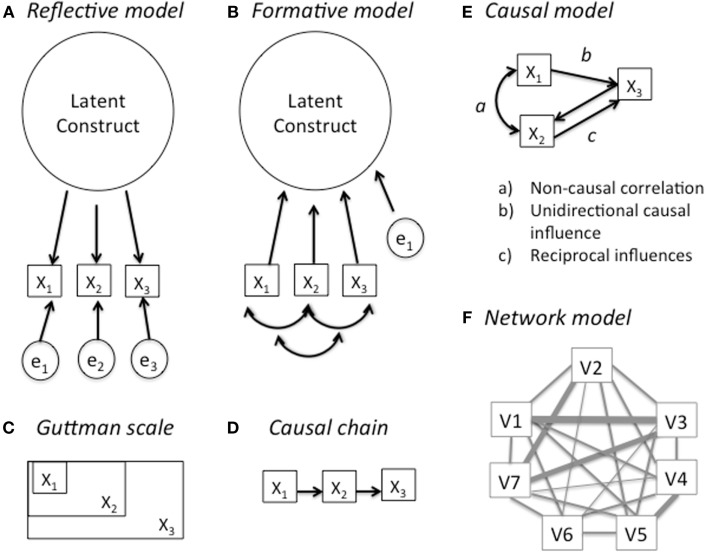
**Alternative theoretical models for interpreting a latent construct**. **(A)** reflective model, **(B)** formative model, **(C)** Guttman scale, **(D)** causal chain, **(E)** causal model, and **(F)** network model.

Traditional factor-analytic models (whether exploratory or confirmatory) are usually specified mathematically as a set of structural equations of the form *X_i_* = *F* + *e_i_*, where each *X* is one of *i* observed or “manifest” variables (e.g., test items or diagnostic criteria), and *F* is the underlying latent construct thought to cause each *X* to take on its observed values [e.g., (9–11)]. Importantly, the *e* terms reflect any idiosyncratic variance associated with the observed variables but not caused by the underlying latent construct of interest. This has an important implication; if any two observed variables share a common latent factor, it is assumed that these variables share nothing systematic in common other than that factor – they are “conditionally independent” unless the default assumption of uncorrelated error terms is explicitly overridden. Any model with these features is now commonly referred to as a “reflective model” (10). The reflective model (see Figure 1A) is a method of constructing unidimensional composite scales and justifying their interpretation as such.

The most common theoretical justification for this interpretation is the *domain sampling* assumption that the observed variables we retain as indicators of the latent construct are essentially interchangeable exemplars sampled arbitrarily from a much larger domain of possible expressions of the construct. “The model of domain sampling conceives of a trait as being a group of behaviors all of which have some property in common. …If the sample [of indicators] we draw from domain is representative, than its statistical characteristics are the same as those of the total domain” [(11), p. 211–212]. Specifically, in expectation any sufficiently large random sample of indicators from the domain should yield the same average value, and the same correlations among indicators. This notion of sampling is of course hypothetical, not literal, and that creates an important conceptual twist: “Instead of specifying a population of some set of entities and then drawing a sample randomly from it, … we have a sample in hand that in turn *implies* a population … having the same characteristics as the sample” [(11), 214 p.].

Most of the published psychometric analyses of DSM criteria that I have examined adopt the reflective model of factor analysis, without explicit justification. But this creates an unacknowledged conceptual puzzle: according to that model, any differences between two criteria – say, withdrawal symptoms vs. interference with important activities – are simply part of the error structure of the model rather than the construct itself, or its composite score. In other words, the distinctive features of each criteria that form the basis for expert debates about DSM construction are actually irrelevant to the model. Under the domain sampling assumption, there should be relatively little to argue about; we can inductively generate large sets of candidate criteria and simply cull out the ones that don’t “load” on the common factor (This is basically how intelligence tests are constructed.). I very much doubt this is how most DSM experts view the diagnostic criterion list, yet this is how the analyses treat it.

### Formative models

There is a less familiar alternative way of specifying a latent factor model – the “formative model” [Figure 1B; see Ref. (9, 10)]. This model is superficially similar – it consists of the same observed indicator variables, plus one or more latent factors, and an error term. But the assumptions are quite different. In a formative model, the latent factor does not cause the observed variables; rather, they cause – or more accurately, “constitute” – the latent factor. Mathematically, the model would be represented by an equation of the form *F* = Σ*X_i_*+*e* but F is now the dependent variable, and there is a single error term for the factor, rather error terms for each observed variable (*X*). That means that anything distinctive or idiosyncratic that distinguishes two observed variables – say, withdrawal symptoms vs. interference with important activities – is part of the construct and its measurement. As a result, formative models are not assumed to be “unidimensional” and indeed, some heterogeneity among the criteria is seen as desirable.

Formative models are not inductive, at least not in the sense that a latent construct emerges from the observed variance of a reflective model. Rather, formative models are a form of “measurement by fiat.” The analyst, or some other authority, decrees that certain observable criteria will collectively constitute what the latent construct actually means. An example is professional accreditation (constituted by education, degree, years of experience, a passing exam score).

A formative model seems to better capture the way many psychiatrists actually debate the DSM criteria, and it also better characterizes the actual decision process – organizational fiat – that determines which criteria are included vs. excluded. But a growing number of simulation studies show that when data actually have a formative structure, fitting them using reflective models can lead to significantly biased and misleading estimates of model fit and factor scores [e.g., (13)]. Whether this helps to explain the puzzle I noted earlier – high unidimensionality despite what are surely conceptually distinct DSM criteria – would probably require focused re-analysis of major DSM data sets^1^.

### A Guttman scale?

There is, however, an alternative psychometric model that might produce unidimensionality despite conceptually distinct measures – a Guttman scale [or a stochastic variant, the Mokken scale; see Ref. (16)]. As suggested by Figure 1C, the variables in a Guttman scale have a cumulative structure. An example might be a diagnosis of AIDS; anyone who has the disease AIDS is infected with HIV, and anyone who is infected with HIV was exposed to HIV at some earlier date when they were still HIV-negative. Thus if we determine that someone is HIV-positive, we can conclude that they were exposed to HIV, but we cannot conclude that they have AIDS or will necessarily have AIDS in the future. Like formative models, Guttman scales can emerge by fiat: with rare exceptions, we decree that those with a Ph.D. must have a Bachelors Degree, and that those with Bachelors Degree must have completed high school. Alternatively Guttman-scaled phenomena can emerge through a chain of causal processes (see Figure 1D) that occur in a consistent order.

If the DSM criteria formed a clear Guttman scale, this might provide a tidy resolution to the puzzle noted at the outset – the fact that psychiatrists argue over the *distinct* features of DSM criteria and yet claim that the DSM provides a *unidimensional* diagnosis of substance use disorder. But the empirical literature is not encouraging. I have only been able to locate two studies that test whether the DSM substance-use criteria form a Guttman scale. Kosten et al. (5) computed Guttman scale scores using DSM-III-R criteria for each of seven substance classes for 83 psychiatric patients. Carroll et al. (2) followed the same procedures using DSM-IV criteria for six substance classes for 521 people drawn from a variety of different clinical and general population sources. Across four substance classes, the Guttman reproducibility coefficients averaged 0.89 for the DSM-III-R study and 0.80 for the DSM-IV study. Common benchmarks for this coefficient are 0.85 or 0.90; diagnoses met the lower standard in both studies for alcohol, cocaine, and the opiates, but not for sedatives, stimulants, or marijuana. More troublingly, if we limit the focus to four criteria that are roughly the same in both version of the DSM – withdrawal, tolerance, “giving up activities,” and “use despite problems” – their relative rankings within a given substance category are inconsistent across the two studies, with correlations ranging from −0.57 to 0.11 (mean *r* = −0.20). Granted, some differences are expected due to differences in year and sample, but it is difficult to see anything like a coherent Guttman measurement model either within or across substance categories.

Another source of evidence comes from comparisons of the prevalence of each criterion, by substance, in different studies. If the criteria come close to forming a Guttman scale, then different studies should find a similar ordering, with the prevalence of some criteria (those near the low end of the scale) being consistently higher than other criteria (those near the high end of the scale). I compared prevalence estimates from three samples (2, 3, 4, 6). The average correlations of the criterion ranks across studies were only 0.54, 0.32, and 0.25 for cannabis, opiates, and cocaine, respectively.

### Causal models and network models

One reason why items have Guttman scale properties is if they have form a simple causal chain (Figure 1F), but the evidence against a Guttman scale interpretation, reviewed above, also casts doubt on any simple causal model. Figure 1E shows a causal model that is more complex than a simple causal chain. Even a cursory examination of the DSM substance-use criteria suggests that they might have this kind of complex internal causal structure. First, many of the criteria require the clinician or the patient to make causal attributions: e.g., “Recurrent use *resulting in* a failure to fulfill important role obligations” or “continued use despite recurrent social problems *associated with* use” (italics added for emphasis).

Second, many of the criteria are likely to have causal linkages to each other. For example, tolerance implies that the user will seek larger doses, which might well increase the time taken to obtain the drug will increase. Withdrawal symptoms and craving have long been implicated in income-generating crime, needle sharing, prostitution, and other forms of physically hazardous and socially dysfunctional behavior [e.g., (17–19)].

Third, in addition to any psychopharmacological mechanisms, most of the criteria are causally influenced by the social, cultural, economic, and legal context in which substance use takes place [see Ref. (20)]. A striking illustration comes from clinical trials for heroin maintenance in Europe (21); when registered addicts are allowed easy access to high-quality heroin, their criminality drops, their health improves, and they are increasingly like to hold a job. But most clients do not quit using; on the contrary, many significantly increase their daily dose, so the intervention reduces their disorder on some criteria while possibly increasing their disorder by other criteria.

Figure 1F, reproduced from Cramer et al. (12), illustrates the kind of elaborate causal network that Borsboom, Kendler, and their colleagues have recently proposed as a more realistic model for many traits. In their framework, latent constructs neither cause observed manifestations (as in a reflective model) nor does an explicit subset of observed variables constitute the latent construct (as in a formative model). Rather, the latent construct is an emergent property of the entire network. An implication of the causal structure in Figures 1E,F is even when simple 1-factor models fit the data, the fit may be spurious in that the model assumed by the equations may be very different than the model that validly describes the processes that generated the data. Moreover, combining them in an “any two of the following” recipe will obscure the valuable information contained in that causal structure.

## Discussion

Judging from past experience, we might expect the next DSM (DSM-6) to surface in about a decade. So in the spirit of constant improvement, I respectfully urge DSM developers to consider pursuing, in parallel, at least three kinds of alternative DSM candidates: a pure reflective model, a pure formative model, and a pure causal network model. One of the three may emerge as superior. But diagnostic systems attempt to serve multiple goals, and it may be advantageous to use different systems for different purposes.

These arguments for greater theoretical and psychometric coherence might seem to have a sort of ivory-tower fastidiousness, if not outright neuroticism. After all, the perfect is surely the enemy of the good, and the DSM does a good job much of the time, at least as judged by the utility that clinicians and managed care organizations seem to find in it.

But I think there are good practical reasons for improving the coherence of the DSM substance use. One is that it might provide a better linkage to drug *policy*. A decade ago, I argued that contemporary thinking about addiction was surprisingly inconsequential for major public policy debates about drug use, or for empirical drug policy analysis (22). The DSM-5 probably helps to close that gap, as it emphasizes the harmful consequences that citizens care about. On the other hand, the gap between the DSM and drug *science* may be growing rather than shrinking. For example, a recent review of seven major scientific theories of drug addiction (23) examines whether each theory can account for various “addictive phenomena.” Of the seven theories, four offer an account of withdrawal and three an account of tolerance – two explicit DSM criteria. Six offer accounts of relapse, and four an account of binging – two phenomena that aren’t directly mentioned in the DSM but are closely related to other DSM criteria. But all seven offer accounts of craving, a criterion that only recently entered the DSM checklist. And four address “sensitization” – which is increasingly recognized as a signature feature of the etiology of addiction but receives no mention in the DSM.

Kosten et al. (5) attribute the unidimensional aspiration behind the DSM to a published WHO memorandum by Edwards (24). Three decades later, that memorandum is still a remarkably insightful analysis. But my reading of it is different than of Kosten et al. While Edwards et al. did argue for a *dimensional* account of dependence, they explicitly rejected the notion that it should be *unidimensional*. Edwards et al. [(24), 233 p.] argue that “what the present model would seem to propose is that a clinical or an operational definition of dependence must be multidimensional and, in terms of measurements, related to a number of phenomena within the syndrome.” And the picture they offered very much seems to anticipate the kind of causal network model that Cramer et al. (9, 12) are developing:

We believe that a system or syndrome model that seeks to take into account of the interaction between drug, person, and environment, is much to be preferred. Any interpretation that places too much emphasis on only one part of the whole system is imperfect and misleading.” [(24), 232 p.].

This essay does not even begin to sketch out what a superior diagnostic system might look like; I don’t even pretend to know. But I am not calling for the abandonment of the DSM-5, or even a change in the list of indicators currently in use. Rather, I am suggesting that we need a better understanding of what the patterns of covariance in DSM data actually mean. If tolerance and withdrawal and craving and psychosocial dysfunction and the other DSM criteria are distinct concepts – and I think they clearly are – why should we expect them to form a single dimension? A system that took seriously their conceptual distinctiveness would facilitate a better understanding of the causal structure that may well link them together. And a system that articulated that causal structure might improve our ability to protect high-risk clients before their problems become severe, and to more closely link treatment decisions to theory and measurement.

## Conflict of Interest Statement

The author declares that the research was conducted in the absence of any commercial or financial relationships that could be construed as a potential conflict of interest.

## Footnotes

^1^An additional explanation for unidimensionality might be rater bias due to shared mental prototypes of dependence among clinicians [compare Ref. (14, 15)].
